# Can Nanotechnology and Materials Science Help the Fight against SARS-CoV-2?

**DOI:** 10.3390/nano10040802

**Published:** 2020-04-21

**Authors:** Maria Chiara Sportelli, Margherita Izzi, Ekaterina A. Kukushkina, Syed Imdadul Hossain, Rosaria Anna Picca, Nicoletta Ditaranto, Nicola Cioffi

**Affiliations:** 1 Chemistry Department, University of Bari “Aldo Moro”, via E. Orabona 4, 70126 Bari, Italy; maria.sportelli@uniba.it (M.C.S.); margherita.izzi@uniba.it (M.I.); ekaterina.kukushkina@uniba.it (E.A.K.); syedimdadul.hossain@uniba.it (S.I.H.); rosaria.picca@uniba.it (R.A.P.); nicoletta.ditaranto@uniba.it (N.D.); 2 IFN-CNR, Physics Department “M. Merlin”, Bari, Italy, via Amendola 173, 70126 Bari, Italy; 3 CSGI (Center for Colloid and Surface Science) c/o Dept. Chemistry, via Orabona 4, 70125 Bari, Italy

**Keywords:** COVID-19, SARS-CoV-2, nanoantiviral, PPE, copper, silver, nanomedicine, contagion, mask, air conditioning

## Abstract

Since 2004, we have been developing nanomaterials with antimicrobial properties, the so-called nanoantimicrobials. When the coronavirus disease 2019 (COVID-19) emerged, we started investigating new and challenging routes to nanoantivirals. The two fields have some important points of contact. We would like to share with the readership our vision of the role a (nano)materials scientist can play in the fight against the COVID-19 pandemic. As researchers specifically working on surfaces and nanomaterials, in this letter we underline the importance of nanomaterial-based technological solutions in several aspects of the fight against the virus. While great resources are understandably being dedicated to treatment and diagnosis, more efforts could be dedicated to limit the virus spread. Increasing the efficacy of personal protection equipment, developing synergistic antiviral coatings, are only two of the cases discussed. This is not the first nor the last pandemic: our nanomaterials community may offer several technological solutions to challenge the ongoing and future global health emergencies. Readers’ feedback and suggestions are warmly encouraged.

**Dear Editor,**

“Are we ready for pandemic influenza?”

With an as-titled article, published in Science in 2003 and heavily cited later, Webby and Webster proposed some interrogatives about the threat of emerging infectious diseases [1]. After the global spread of severe acute respiratory syndrome caused by a novel coronavirus (SARS-CoV) in 2002 and the H5N1 (avian) influenza virus in the 2003, the scientific community tried to set some medical key points to better respond to potential future pandemic diseases. The demand for a sufficiently large supply of antiviral drugs and vaccines was clear. Other infectious diseases arose over the years, including the pandemic H1N1 influenza (swine flu), the Middle East respiratory syndrome coronavirus (MERS-CoV), and the current pandemic, named coronavirus disease 19 (COVID-19).

In late 2019, a novel pneumonia caused by an unknown pathogen emerged in the city of Wuhan, central China [2]. After few months, and specifically on 19 April 2020, the pandemic counts more than 2,200,000 contagions around the world, according to the World health Organization (WHO) daily reports [3].

Currently, many different antiviral agents, including re-purposed drugs, are under testing in clinical trials to assess their efficacy against the new virus, but the quest for an effective treatment against COVID-19 is still open [4,5,6,7].

Different strategies were pursued, to treat coronavirus related to MERS and SARS infections, including the use of inhibitors of viral and host proteases, interferons (IFNs), and host-directed therapies. One of the first antiviral drugs tested, the ribavirin, a nucleoside analog that acts as RNA polymerase inhibitor [8], was used on patients with SARS and MERS [9]. However, ribavirin monotherapy had limited activity against SARS-CoV and led to significant hemolysis collateral effect [10]. Ribavirin is more commonly used together with lopinavir, ritonavir and HIV protease inhibitors [11]. These combinations were demonstrated to improve the outcome of patients with SARS [9].

Based on antiviral treatment against previous coronavirus SARS and MERS, lopinavir/ritonavir combination was tested [12] too. There are also signs of the benefit of both interferon-α (IFN-α) and interferon-β (IFN-β) treatments [13]. In addition, some studies in vitro and in non-human primates’ trials demonstrated that the combination treatment with ribavirin and INFs improves clinical outcomes in MERS-CoV infection [14]. Translating the findings from these studies into clinical trials remains of particular importance, especially taking into account drugs availability, pharmacokinetic properties, and possible side effects, including long term or permanent ones.

The vital role of the spike protein (S protein) of coronaviruses makes this glycoprotein an important therapeutic target, because it guides coronavirus entry into host cells, by providing the binding and the fusion of virus on the host cell membrane. S protein is composed of two subunits: S1 recognizes and binds to host receptors, and S2 facilitates fusion between the viral envelope and the host cell membrane [15]. Numerous studies have explored how to target this first stage of the virus lifecycle. These methods mainly involve peptidic fusion inhibitors, anti-CoV neutralizing monoclonal antibodies, and entry receptor antagonists. However, none of these curative agents is approved for commercial use in humans [16]. Regarding the SARS-CoV-2 infection, in the absence of a clinically proved effective antiviral therapy against COVID-19, a combination of different drugs has been often supplemented (Table 1).

Remdesivir, an adenosine analogue, is a broad-spectrum antiviral with potent in vitro efficacy against multiple genetically unrelated RNA viruses [20]. It is currently under clinical development for the treatment of Ebola virus infection and it also demonstrated a good antiviral result in vitro against SARS and MERS coronaviruses [6]. Remdesivir also revealed as an effective antiviral agent in vitro against COVID-19 infection [21]. Among small-sized molecular agents approved for viral human diseases, an immune modulator, chloroquine, shows inhibitory effects against SARS-CoV-2 [19]. It is known as a potential broad spectrum antiviral drug, widely used as anti-malarial drug [22]. Chloroquine (CQ) blocks virus infection by increasing the endosomal pH required for the fusion between virus and cell. It also interferes with the glycosylation of cellular receptors of SARS-CoV [5,22]. A recent in vitro study demonstrated that CQ functions at both entry- and at post-entry-stages of the SARS-CoV-2 infection [19,23]. Besides its antiviral activity, CQ also presents immune-modulating activity, which can enhance its antiviral effect in vivo. Notably, CQ could be replaced by the less toxic hydroxychloroquine (HCQ) [23].

In this scenario of non-univocal medical treatments and vaccine unavailability, it is evident that, besides the population above 75-year-old and people with immunodeficiency, or chronic and oncological diseases, the category that is most exposed to COVID-19 is that of hospital personnel [24]. It is imperative to ensure the protection of health-care workers. This is not only crucial to guarantee continuous patient care (in all nosocomial environments), but also to prevent virus transmission by unaware SARS-CoV-2 carriers [25]. Besides, contagion protection for the whole population is mandatory in all those cases when social confinement is not possible due to force majeure (work, health problems, caregiving, etc.). Finally, in the first stages of the removal of confinement measurements, it is envisaged that we will all need safer and more effective personal protective equipment (PPE).

Contagion rates of COVID-19 are much higher than those reported for the well-known SARS [26]. This brought to the fast diffusion of the virus in all continents [27,28]. COVID-19 can easily propagate via cough or respiratory droplets, contact with bodily fluids, or from contaminated surfaces (Figure 1). Secondary infection routes involve touching dirty/contaminated surfaces, followed by self-inoculation of mucous membranes. A study carried out on 29 patients [29] estimated that secondary routes are less probable than direct contagion (including eye-, nose-, oral-, etc.). Nevertheless, this risk is not considered negligible by different other sources, including WHO [30]. Many commonly touched surfaces, along with nosocomial environments and PPE have been tested against this risk. Based on a previously published paper [31], which revealed the persistence of human coronavirus 229E on the surface of common materials, G. Kampf recently reviewed the topic for SARS, MERS and SARS-CoV-2 viruses [32,33].

Surface contamination has recently been found to be more significant than originally thought in the spread of this viral disease. In fact, despite the use of proper PPE [34], health-care workers continued to contract SARS-CoV-2, even after barrier precautions were widely implemented. The reason behind this was found in the air, environmental, and PPE contamination in hospitals [35]. Besides common touching surfaces (tables, beds, door handles, light switches, toilet sites, etc.), even anteroom floors and air fans outlets were found to be contaminated [36]. The latter fact is explained by the persistence of SARS-CoV-2 in aerosols, up to several hours [37].

Many resources and biotechnological capabilities are currently directed towards the development of vaccines and treatments against COVID-19. Anyway, given the significance of the surface and air contamination in the spread of the virus, attention should be paid also to the development of antiviral and antibacterial surfaces, along with decontamination equipment and technologies.

We hypothesize that using proper air filtering may reduce the viral load in the environment, sufficiently to decrease the probability of health-care worker infection through flaws in PPE, or in confined public transportation [37]. According to a study of the Purdue University (West Lafayette, IN, USA) [38], air conditioning (AC) systems are not designed to filter out particles as small as the coronavirus. Consequently, the disease could rapidly circulate to other individuals in closed communities as it presumably happened, as an example, in cruise ships [39].

Inverse computational fluid dynamics (CFD) models are available to identify the spread of air particles in passenger vehicles [40]. CFD models, and the airborne nature of SARS-CoV, could be considered to understand the spread of coronavirus in airplane passengers [41].

High-Efficiency Particulate Air (HEPA) filters can limit the spread of airborne fungi, viruses, and bacteria [42,43]. Their use could further benefit from the implementation of a new generation of safe and effective multifunctional antibacterial and antiviral agents.

The contamination of latex/nitrile gloves, N95 respirators, hospital scrubs, overshoes, and floors in a nosocomial environment is considered a serious issue [35,44,45], because it helps the uncontrolled spreading of the disease: health-care workers are anxious about passing the infection to other patients and to their families [46].

Besides nosocomial environments, the contamination of surfaces can be regarded as responsible for many other contagion episodes. As an example, Australian Health Authorities have recently confirmed several cases of COVID-19 among baggage handlers. The circumstances gave rise to concerns about cleaning the luggage and about the virus survival time on hard, smooth materials, such as plastic and metal. The detailed explanation of contamination sources and the current number of contagions among baggage handlers worldwide are still unknown. However, extensive cleaning and disinfecting procedures were implemented in international airports, to reduce the surface contamination of luggage [47].

More generally, many disinfection protocols have been developed so far, which mainly involve the use of sodium hypochlorite [35], 70–85% ethanol [33,45,48], iodine-based and quaternary-ammonium-salt-based disinfectants [32,48,49,50,51]. Van Doremalen et al. [36] tested the viability of SARS-CoV-2 in different environmental conditions (aerosols, plastic, stainless steel, copper, and cardboard). The longest viability was on stainless steel and plastic surfaces; the estimated median virus half-life being approximately 5.6 h on stainless steel and 6.8 h on plastic. Copper was found to be effective in inactivating the virus in a shorter time. These findings are in agreement with what already reported on CoV-229E in 2015 [31]: brasses containing at least 70% copper were very effective at inactivating CoV-229E, and the rate of inactivation was directly proportional to copper percentage. Copper ion release and the generation of reactive oxygen species (ROS) were demonstrated to be responsible for the inactivation of coronaviruses on copper and copper alloy surfaces [36]. Several already-approved biocides based on silver or zinc oxide are just waiting to be tested against SARS-CoV-2 as well.

Based on these findings, we make a heartfelt appeal to the (Nano)Materials Science community: we can exploit the well-known antimicrobial properties of formulations and nanostructures containing copper, silver, and zinc species [52,53] to fight COVID-19 and, more specifically, to prevent and limit both contamination and contagion.

Specifically, the use of copper salt nanoparticles and/or solutions (chloride, iodide, sulfide, etc.), which are known for having an antiviral effect [54,55,56,57,58], could be helpful in the development of PPE with improved shielding properties. As an example, the treatment of non-woven overshoes, surgical gowns, hair cups, respirators, etc. with copper ions could help in preventing the unwanted nosocomial virus spreading by medical personnel. In principle, the presence of metal ions could reduce (or set to zero) the viability of CoV on these substrates, which can be considered as simple carrier interfaces for the infection spread. Analogously, the treatment of common touching surfaces with Cu, or the use of copper brasses for all those surfaces which need to be kept sterile, could be extremely helpful.

Nanotechnology can offer a great support in the design of contamination-safe equipment in this era of pandemic diseases. Metal-loaded nanocomposites are known to be extremely effective in all those cases in which a controlled and long-lasting ionic release is required [59,60]. The controlled release of ionic copper is the key to tune the antimicrobial and antiviral properties of surfaces [61,62].

Metal nanoparticles can act as ion reservoirs for the controlled release of bioactive ions, thus tuning the production of ROS species too. The embedding of metal nanoparticles (copper ones, in principle) in polymer matrices could help in the tuning of metal release properties, and at the same time in minimizing the risk of nanoparticle release into the environment [53]. Moreover, very simple and reliable routes to synergistic nanoparticles combining a copper core and a quaternary ammonium shell (both of which are capable, in principle, of expressing a strong antiviral action) are available in recent literature [63].

To the best of our knowledge, there are few publications about the treatment of nosocomial PPE with copper nanoparticles or copper oxides and salts. It is evident that inadequate PPE and inappropriate PPE guidelines can be responsible for the death of many health-care workers and for viral nosocomial spreading. Bhattacharjee and co-workers reviewed the topic in 2019, before the spreading of SARS-CoV-2, taking into account other viral pandemic diseases like Ebola, SARS, and MERS [64]. They reported that metal-grafted graphene oxide (GO), for the modification of non-woven tissues, showed to have very effective antimicrobial properties. Graphene derivatives have been reported to be used as antimicrobial composites with different metals (Ag, Fe, Cu, Zn, etc.), and photocatalysts (TiO_2_, CdS, MnS_2_, etc.) [65]. GO grafted with metal nanoparticles has been investigated as a potential treatment for PPE [66]. Specifically, it is known that silver and copper nano-based systems loaded on GO are very effective against both enveloped and non-enveloped viruses [65,67]. Anti-influenza copper oxide-loaded polypropylene respirators were proposed in 2010; Borkow et al. demonstrated that copper oxide-impregnated masks safely reduced the risk of influenza virus environmental contamination, without altering the filtration capabilities of the masks [68]. However, no reports about the impregnation of masks and other PPE with copper salt solutions were found in literature [68,69]. Their use is actually part of our future research plans, which will involve both nanophases and reference compounds and salts. In order to prevent the potential nano-toxicity of metal nanoparticles on masks and respirators through inhalation, we think that, besides embedding nanoparticles in polymer matrices (vide infra), the best strategy could be the use of copper salts for the impregnation of PPE pieces. In fact, when used at low concentration levels, Cu(II) ions have low cytotoxicity on eukaryotic cells [70].

Recently, polyurethane/CuO nanocomposites were developed, acting as an effective antimicrobial filter for air purification. It is worth noting that microparticles of CuO are a more suitable additive for the modification of polyurethane filters than nanoparticles [71], thus reducing nanotoxicity risks. A recent example of an antiviral air filtering system for transportation was based on the use of SiO_2_-Ag NPs as active material against MS2 bacteriophage [72].

The importance of Nanotechnology in fighting viruses is not enough explored. One of the possible directions of further investigation is related to the use of nanomaterials to fight virus resistance to conventional therapies; this resistance can be due to the accelerated virus adaptation in peripheral protein sequence, thus resulting in the development of a new viral strain [73].

The antiviral action of metal nanoparticles, notably of silver nanoparticles (AgNPs), is well known. They act as viral reproduction inhibitors, and their viricidal activity depends on the target virus. For example, the AgNP ability to inhibit the viral entry in host cells, in the case of the HIV-1 virus, was reported, demonstrating that AgNPs are able to interact with cell receptors [73]. Regarding double strain RNA (dsRNA) viruses, AgNP interaction with a viral genome was found, inhibiting viral replication [73]. Likewise, gold nanoparticles (AuNPs) stabilized by biocompatible polymers showed antiviral activity against HIV-1 and some subtypes of influenza virus (e.g., H1N1, H3N2, H5N1) [73].

It was also demonstrated that gold nanoparticles coated with sulfated ligands, silver nanoparticles, and hybrid silver-copper nanoparticles are able to bind the HIV envelope glycoprotein gp120 and to inhibit in vitro HIV-1 infection in cellular models [74,75]. In addition, a size-dependent interaction with HIV-1 (size in the range of 1–10 nm) was reported [75]. It was also demonstrated that functionalized AgNPs (e.g., with tannic acid and mercaptoethane-sulfonate) may have the ability to prevent HSV infection by the direct inhibition of virus attachment, penetration and post-infection spread [76,77]. Single-walled carbon nanotubes (SWCNTs) were also proposed as antiviral carriers. Specifically, isoprinosine and ribavirin were chemically linked on the SWCNT surface to carry drugs across biological membranes; then, the increment of the antiviral activity of hybrid materials, compared to that of antiviral drugs, was tested [78,79].

It is known that very small metal NPs (<10 nm) are able to go through the cell membrane and inhibit post-attachment virus replication [80]. As an example, it was recently reported that a nanotechnology-based solution containing titanium dioxide and silver ions was used for street disinfection in Milan, Italy [81].

Given all these premises, the general question is: can inorganic nanoparticles be useful in affecting the early viral lifecycle stages?

The specific question is: is it possible to realize active nanomaterials able to inhibit the binding and fusion of viruses on the host cell?

We believe that it is certainly possible to find a nanotechnological solution for these quests.

Our idea is to realize hybrid antiviral nanomaterials by functionalizing metal nanoparticles with typical antiviral drugs, with enhanced synergistic efficacy. These synergistic nanoantivirals might offer a great help in increasing the efficacy of PPE and improving the safety of common touch surfaces.

They can be used to modify surgical masks and respirators, which are crucial targets in the fight against virus diffusion [82]. Among the WHO-approved antiviral drugs, CQ and HCQ demonstrated their ability to act in the first step of viral lifecycle. CQ and HCQ are aminoquinolines, a class of heterocyclic scaffolds with an amino group. They are able to form metal complexes with Fe(II), Ni(II), Zn(II), and Cu(II) ions [83]. In addition, metal complexes have been investigated as drug delivery agents, leading to reduced side effects and improved pharmacokinetics. It is worth noting that metal complexes of 8-hydroxyquinoline have been already shown to be antiviral agents [84].

Hence, we believe that CQ and HCQ could be proficiently used to functionalize metal nanoparticles. Other antiviral/metal nanostructure combinations could be possible, as well.

This is of course our perspective viewpoint and needs to be tested. *We warmly encourage the readers working in this area to investigate the development of hybrid antiviral metal-CQ/HCQ nanomaterials. The resulting synergistic nanoantivirals could be very useful to prevent the diffusion of the virus. They could be implemented in coatings for hard surfaces and used as PPE-modifiers to increase their protective action.*

At the present stage, we think that sharing these ideas and triggering our community to develop a deeper level of knowledge about the (unexplored) antiviral properties of nanomaterials could be a good way to fight this pandemic.

It is likely that nanotechnology has enormous potential in the prevention, diagnosis, and treatment of COVID-19. In this communication, we do not cover the diagnostics and treatment outcomes, although we certainly quote the considerable perspectives in (i) designing sensors for developing quick-response COVID-19 tests [85], and (ii) developing novel nanomedicines or theranostic tools [86]. In the future, we could also envisage the development of nanomedicines combining biodegradable nanocarriers [87,88] and one of the aforementioned (nano)antiviral agents, for possible aerosolization in the lung of patients under ventilation. This would allow for a much higher local concentration, i.e., a better efficiency, while limiting the penetration in the bloodstream and thus the side effects.

Reverting to the prevention stage, we strongly believe that contagion-safe PPE (from respirators to surgical gowns, overshoes, hair cups, etc.) and nanotechnology-enabled highly effective antiviral disinfectants, can be considered the most effective way to prevent viruses from spreading.

The readers working in the nanotechnology area are warmly encouraged to contact us with their comments or proposals. Sharing our ideas and setting common actions against this pandemic will be the key to success.

The present pandemic is not the first, nor will it be the last one. As researchers working on nanomaterials for the life sciences, we need to ensure that we have tools in place to deal with the COVID-19, as well as with any future pandemics.

Nanomaterial-based antiviral and antibacterial textiles, non-woven disposable products, packaging solutions, antiviral coatings, synergistic/multifunctional surfaces, air-conditioning filters, PPE, are just few of the possible examples requiring our prompt technological answer. While many good research papers have been published on metal nanoparticles to be used as antibacterial or antiviral agents, the commercialization of novel functional nanomaterials still appears to be limited by nanotoxicology concerns or by several other practical aspects (off-target and/or any other unpredictable effects, costs, production yield, durability, environmental impact, etc.). This is a good time to ask why and how we can facilitate the turning of these research projects into safe and viable products.

In the field of nanoantimicrobials, we have always pursued the use of bioactive nanoparticles as water-insoluble, polymer-confined nano-reservoirs, providing a source of ionic release, without being released as entire nanophases in the contact matrices (e.g., physiological solutions, food, sweat, humid air filtered through air-conditioners, etc.) [52,62].

Blocking the nanoantivirals in an adequately stable embedding matrix could be, again, the right way to ensure safety, preventing *a priori* nano-toxicological risks. Other technological solutions need to be envisaged in order to promote nanosafety and support technological knowledge transfer and commercialization. This is becoming more and more a part of our academic mission [89,90].

We encourage our nanomaterials community to actively exploit its impressive nanotechnological background to challenge the ongoing global health emergency.

## Figures and Tables

**Figure 1:**
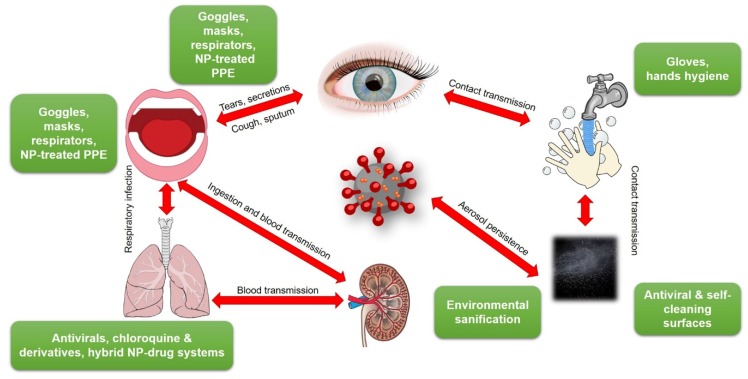
Transmission pathways of SARS-CoV-2 and possible containment means. NP = nanoparticle; PPE = personal protective equipment. This figure was created using images under the Creative Commons licenses. This figure is a derivative of: “Human anatomy” by Wikimedia Commons, used under CC; “SugarandSkullDesigns” by Pixabay, used under CC; “Blubberfisch“ by Pixabay, used under CC; “Shutterstock” by FreeSVG, used under CC-BY; “OpenClipart” by FreeSVG, used under CC-BY; “James Gathany” CDC Public Health Image library used under CC-BY.

**Table 1:** Non-exhaustive list of the most common antiviral agents used to fight coronavirus disease 19 (COVID-19).

Antiviral Drug	Virus Infection	Action Mechanism	References
Ritonavir/Lopinavir	HIV, SARS, MERS	Protease inhibitors, they are usually used in combination with other drugs.	[12]
Remdesivir	Ebola, SARS, MERS	Pre-mature termination of RNA.	[6,12]
Arbidol	A and B influenza, hepatitis C, SARS	Blocking viral fusion.	[17]
Chloroquine and Hydroxychloroquine	Malaria	Blocking virus infection.	[17,18,19]
